# Role of arousal in diagnosing sleep apnea in atrial fibrillation patients

**DOI:** 10.1007/s11325-025-03435-8

**Published:** 2025-08-07

**Authors:** Susana Sousa, Carlos Teixeira, Dina Grencho, Sara Dias, Marta Drummond, António Bugalho

**Affiliations:** 1 Sleep Unit, CUF Tejo Hospital and CUF Descobertas Hospital, Lisbon, Portugal; 2 Pulmonology Department, CUF Tejo Hospital, Lisbon, Portugal; 3 NOVA Medical School, Lisbon, Portugal; 4 Comprehensive Health Research Center (CHRC), Lisbon, Portugal; 5 Nox Research, Nox Medical, Katrínartún 2, 105 Reykjavík, Atlanta, Iceland; 6 H²M - Health and Human Movement Unit, Nova de Famalicão, Polytechnic University of Health, CESPU, Gandra, Vila Portugal; 7 Lisbon School of Medicine, Lisbon, Portugal; 8 Sleep and Non-Invasive Unit, Centro Hospitalar e Universitário São João, Porto, Portugal; 9 Faculty of Medicine, University of Porto, Porto, Portugal

**Keywords:** Arousal, Atrial fibrillation, Hypopneas, Polysomnography, Scoring, Sleep apnea

## Abstract

**Introduction:**

Obstructive sleep apnea syndrome (OSAS) is highly prevalent in patients with atrial fibrillation (AF) and may influence rhythm control outcomes. Accurate diagnosis is essential but depends on the criteria used to define respiratory events. This study aimed to evaluate how the inclusion of EEG arousals in hypopnea scoring affects the diagnosis and severity classification of OSAS in patients with AF.

**Patients and methods:**

We conducted a prospective analysis of 88 consecutive patients with AF (paroxysmal or persistent) referred for sleep evaluation with ambulatory type II polysomnography (PSG). Hypopneas were scored according to two criteria: [1] ≥ 3% oxygen desaturation, and [2] ≥ 3% desaturation or EEG-defined arousal. Apnea–hypopnea index (AHI) and OSAS severity were compared across both definitions.

**Results:**

Participants had a mean age of 63 ± 9.7 years, were predominantly male (68%), and had a mean BMI of 30 ± 4.8 kg/m². OSAS was diagnosed in 100% of patients. Using the desaturation-only criterion, OSAS severity was classified as mild in 24.7%, moderate in 31.8%, and severe in 43.5% of patients. In contrast, scoring hypopneas based on desaturation or arousal led to reclassification: 5.7% mild, 17.0% moderate, and 77.3% severe. Thirty-one patients classified as severe OSAS were missed using desaturation-only scoring. The number of hypopneas detected was significantly higher when arousals were included (200.0 ± 105.6 vs. 81.9 ± 48.9; *p* < 0.001), with a moderate positive correlation between the two methods (*r* = 0.436).

**Conclusion:**

The use of arousal-inclusive criteria significantly increases OSAS detection and alters severity classification in patients with AF. Relying solely on oxygen desaturation may lead to underdiagnosis and misclassification, particularly in non-desaturating patients. Incorporating EEG arousals into hypopnea scoring provides a more accurate assessment of disease burden and may support more effective, individualized treatment strategies.

## Introduction

Obstructive Sleep Apnea Syndrome (OSAS) is highly common in patients with atrial fibrillation (AF) and can reduce the success rate of rhythm control interventions [1]. Current patient assessment requires a thorough clinical evaluation, the use of sleep questionnaires, and an appropriate sleep study to confirm the diagnosis of sleep-disordered breathing (SDB). However, the most effective method for screening SDB in patients with AF remains undetermined.

According to the International Classification of Sleep Disorders, diagnostic criteria for OSAS in adults, require either: (i) five or more predominantly obstructive respiratory events per hour of sleep during polysomnography (PSG) or per hour of monitoring time during a home sleep apnea test (HSAT), accompanied by symptoms or comorbidities, or (ii) fifteen or more predominantly obstructive respiratory events per hour of sleep during PSG or per hour of monitoring time during HSAT. Respiratory events can include apneas, hypopneas, or respiratory effort-related events. The American Academy of Sleep Medicine (AASM) is responsible for defining the scoring criteria for these features. As definitions of respiratory events has evolved over time alongside technological advances, the current version of AASM Manual for the sleep and associated events scoring defines hypopnea as a ≥ 30% drop in peak respiratory excursion from baseline, lasting ≥ 10 s, combined with either a ≥ 3% oxygen desaturation from baseline or an EEG-detected arousal [2].

Given that OSAS is a highly prevalent condition in the AF population, there is a clear need for a simple, cost-effective screening method suitable for large-scale use. Although simplified tests, as HSAT, are more accessible and convenient, allowing for AHI assessment based on recording time, they lack sleep staging and arousal detection capabilities. As a result, they may underestimate disease severity by missing critical information.

An arousal is defined as an abrupt shift of the EEG frequency, including alpha, theta and/or frequencies greater than 16 Hz (excluding spindles) lasting at least 3 s, and preceded by at least 10 s of stable sleep [2]. These cortical arousals may occur spontaneously or be triggered by SDB and can affect heart rate, blood pressure, and cardiac dynamics [3].

Clinically, overnight PSG remains the gold standard for evaluating arousal frequency and cause. The arousal index (AI), which quantifies the number of arousals per hour of sleep, is widely used to assess sleep fragmentation and has been associated to changes in cardiovascular parameters, regardless of the underlying mechanism [4].

Historically, the 1999 AASM recommended criteria for hypopnea scoring required an oxygen desaturation of > 4% and did not consider arousals [5]. The 2012 revision expanded these criteria to encompass either a ≥ 3% oxygen desaturation or an EEG arousal [6]. This change for scoring hypopneas increased diagnostic sensitivity for clinically relevant respiratory events during sleep, but also affected how AHI is calculated.

This study aimed to assess the diagnostic and clinical implications of incorporating arousals into hypopnea scoring in a real-world cohort of AF patients undergoing type II polysomnography.

## Materials and methods

### Study participants

Consecutive patients aged between 18 and 75 years, with a diagnosis of AF were evaluated during cardiology outpatient consultations and referred for sleep assessment and PSG. Exclusion criteria included a prior diagnosis of OSAS, unstable coronary artery disease, myocardial infarction, or percutaneous coronary intervention within the three months preceding enrollment.

Participants were recruited at CUF Tejo Hospital between January 2 and November 3, 2023. The study was approved by the local ethics committee, and written informed consent was obtained from all participants.

### Study protocol

All participants underwent a 12-lead electrocardiogram to confirm AF diagnosis and were subsequently referred for sleep assessment using a type II ambulatory PSG recording system.

Ambulatory PSG recordings were performed with the Nox A1 PSG system (Nox Medical, Reykjavik, Iceland). The following signals were recorded simultaneously: 6-channel electroencephalography (leads: F3A2, F4A1, C3A2, C4A1, O1A2, O2A1), left and right electrooculography (LOC, ROC), and submental electromyography (3 unipolar leads). Additional signals included nasal airflow pressure (measured via nasal cannula), thoracic and abdominal respiratory effort (assessed with thoracic and abdominal inductive plethysmography), and snoring (recorded with a built-in microphone). Body position and activity were monitored using an internal 3-axis accelerometer (± 2 g, 10 Hz sampling frequency), and both oximetry and sound intensity (dB) were recorded.

All PSG data were manually scored by a certified and experienced sleep technologist according to the AASM scoring guidelines [2], serving as the reference standard. The AHI was defined as the total number of apneas and hypopneas per hour of sleep. Hypopneas were defined as a ≥ 30% reduction in airflow lasting for at least 10 s, associated with either a ≥ 3% oxygen desaturation or an EEG-defined arousal, in accordance with current AASM criteria.

Subsequently, we retrospectively rescored the original PSG recordings using only the physiological signals typically available in a level III polygraphy setup, namely: respiratory effort (thoracic and abdominal belts), airflow (nasal pressure), pulse oximetry and body position, while excluding EEG and other neurophysiological channels. In this re-evaluation, AHI was defined as the combined number of apneas and hypopneas per hour of sleep, with hypopneas identified as a 30% reduction in airflow for at least 10 s, followed by a 3% desaturation.

Data analysis was conducted using the Statistical Package for Social Sciences (SPSS), version 28. Descriptive statistics (mean, standard deviation, median and interquartile range) were calculated for all variables. Bivariate analysis, including Mann-Whitney, t-student and chi-square tests were used to assess gender differences. The level of statistical significance was set at *p* < 0.05.

## Results

A total of 88 patients with AF with no previous diagnosis of sleep apnea were included in the study. The mean age was 63 ± 9.7 years, and 68% were male. The mean body mass index (BMI) was 30 ± 4.8 kg/m². All patients completed an ambulatory type II PSG with a median sleep efficacy of 83.5% ± 14.1. The clinical characteristics of the study population are summarized in Table 1.

**Table 1:** Baseline characteristics of the study population

Characteristic	Value
Sex	
Male, N (%)	60 (68.2)
Female, N (%)	28 (31.8)
Age, years (mean ± SD)	63 ± 9.7
BMI, Kg/m^2^ (mean ± SD)	30 ± 4.8
Hypertension, %	46.5
Diabetes, %	17.85
Epworth Somnolence Scale, mean ± SD	6.3 ± 4.386
Snoring, %	64.7

Legend: BMI, Body Mass Index; SD, standard deviation

Our study revealed a 100% prevalence of OSAS in this specific AF population. Using the combined criterion of ≥ 3% oxygen desaturation or EEG arousal for scoring hypopneas, the AHI indicated that 5.7% of participants had mild OSAS, 17.0% had moderate OSAS, and 77.3% had severe OSAS. In contrast, when hypopneas were scored using oxygen desaturation only, the distribution shifted to 24.7% mild, 31.8% moderate, and 43.5% severe OSAS (Table 2). This discrepancy highlights a substantial underestimation of disease severity when arousals are excluded from scoring, with 31 cases of severe OSAS not identified using desaturation-only criteria.

**Table 2:** Comparison of OSA severity based on AHI using different hypopnea definitions (desaturation only vs. desaturation and arousal)

AHI	OSAS severity		
	Mild *N* (%)	Moderate *N* (%)	Severe *N* (%)
Hypopnea desaturation and arousal criteria	5 (5.7)	15 (17.0)	68 (77.3)
Hypopnea desaturation criteria	21 (24.7)	27 (31.8)	37 (43.5)

Legend: AHI, Apnea Hypopnea Index; OSAS, Obstructive sleep apnea syndrome

There was a statistically significant difference in the number of hypopneas identified by each method (*p* = 0.000847), with a moderate positive correlation between the two approaches (*r* = 0.436) (Table 3). On average, the number of hypopneas detected with the desaturation-only criterion was 81.9 ± 48.9, compared to 200.0 ± 105.6 when both desaturation and arousal criteria were used. The application of hypopnea criteria requiring both oxygen desaturation and arousal resulted in a marked shift toward higher OSA severity classification, with 94.3% of patients categorized as having moderate to severe OSAS, compared to only 75.3% when using desaturation-only criteria. Additionally, when OSAS severity was dichotomised into mild versus moderate-to-severe categories, the use of the desaturation-only criterion classified 24.7% of patients as mild, whereas this proportion decreased to 5% when arousals were included (Table 4). Importantly, three patients were classified as AHI < 5 when using the desaturation-only criterion, underscoring the potential for complete diagnostic oversight in certain cases. Furthermore, when we performed a subgroup analysis by gender and compared the proportion of increase in AHI between men and women, with and without the inclusion of arousal criteria in AHI scoring, we found that the statistically significant difference persisted: *p* = 0.002 (male) and *p* = 0.03 (female) (Table 5).

**Table 3:** Comparison of hypopneas per definition criteria (desaturation only vs. desaturation and arousal)

Hypopneas	Mean ± Standard Deviation	Correlation Coefficient	*p*-value
Desaturation only	81.92 ± 48.86	0.436*	< 0.001
Desaturation and arousal	200.02 ± 105.60		

**Table 4:** Comparison of OSA severity based on AHI using different hypopnea definitions (desaturation only vs. desaturation or arousal)

	OSAS severity	
AHI	Mild N (%)	Moderate-to-severe N (%)
Hypopnea desaturation and arousal criteria	5 (5.7)	83 (94.3)
Hypopnea desaturation criteria	21 (24.7)	64 (75.3)

Legend: AHI, Apnea Hypopnea Index; OSAS, Obstructive sleep apnea syndrome

**Table 5:** Comparison of OSA severity based on AHI using different hypopnea definitions (desaturation only vs. desaturation or arousal) according to gender

Male		*N*	%
Hypopnea desaturation and arousal criteria	Mild	1	1.7
	Moderate-Severe	49	98.3
Hypopnea desaturation criteria	Mild	13	21.7
	Moderate-Severe	47	78.3

Female		*N*	%
Hypopnea desaturation and arousal criteria	Mild	4	14.3
	Moderate-Severe	24	85.7
Hypopnea desaturation criteria	Mild	8	32.0
	Moderate-Severe	17	68.0

Regarding treatment, all patients with AHI ≥ 15 were proposed to initiate Continuous Airway Positive Pressure (CPAP) therapy, while those with mild forms of the disease were managed based on the severity of their sleepiness. Importantly, the identification of a greater number of patients classified as moderate to severe, due to more accurate hypopnea scoring, directly influenced treatment decisions in this population.

These findings indicate that scoring criteria significantly influence OSAS diagnosis and severity classification, with implications for clinical decision-making and treatment planning in AF patients.

## Discussion

This prospective study evaluated the impact of different hypopnea scoring criteria - oxygen desaturation alone versus desaturation or EEG-detected arousal, on the diagnosis and severity classification of OSAS in patients with AF.

Our findings revealed a 100% prevalence of OSAS among consecutive AF patients, a markedly higher rate than previously reported in the literature [7]. The prevalence of OSAS in AF patients varies considerably, ranging between 21 and 82% [8–11]. The high prevalence observed in our study is likely attributable to the comprehensive diagnostic capabilities of type II PSG and the use of broader inclusion criteria, rather than selective recruitment of symptomatic individuals Other studies often rely on simplified diagnostic methods, such as HSAT or level III devices, which may fail to detect non-desaturating but clinically significant events and often underestimate prevalence due to the inability to detect arousals [12, 13].

OSAS diagnosis is currently based on the AHI, which quantifies the frequency of respiratory events (including apneas and hypopneas) per hour of sleep [14]. Disease severity is determined not only by the event rate, but also by their immediate physiological consequences, including oxygen desaturation and sleep arousals. The 2012 AASM criteria, which include EEG arousals alongside oxygen desaturation in the definition of hypopneas, enhance the sensitivity of diagnostic assessments, ensuring more comprehensive case identification and more precise classification of OSAS severity [6]. Our findings demonstrated that inclusion of EEG arousals in hypopnea scoring led to significant reclassification of OSAS severity, identifying 31 additional cases of severe OSAS that would have been misclassified using desaturation-only criteria. This emphasizes the limitations of simplified scoring methods, particularly in non-desaturating patients.

Cortical arousals, though often not accompanied by oxygen desaturation, are physiologically significant. Repetitive arousals have been implicated in heightened sympathetic activity and autonomic imbalance. The sleep fragmentation index, closely linked with arousals, has shown to strongly correlate with low frequency/high frequency ratio and very low frequency heart rate variability components, established markers of sympathetic overdrive in SDB. Chronic sympathetic activation may promote atrial remodelling, fibrosis and altered ion channel function, thereby contributing to AF development and maintenance [15, 16]. Importantly, respiratory-related arousals may not result in significant oxygen desaturations but still impose substantial autonomic stress, characterized by abrupt sympathetic activation. Consequently, hypopnea scoring that omits arousals risks overlooking events with direct arrhythmogenic potential. This neurophysiological response is critical in patients with AF, where sympathetic activation plays a critical role in atrial instability [15]. Shahrbabaki and colleagues demonstrated the importance of sleep arousal burden, namely the association between arousal burden and all-cause and cardiovascular mortality, and the importance of identifying arousals in older men and women in risk stratification [17]. This reinforces the clinical importance of integrating arousal scoring into standard diagnostic pathways, especially in high-risk populations such as patients with AF as an independent predictor of cardiovascular and all-cause mortality, beyond AHI or oxygen desaturation index (ODI) values. Therefore, accurate identification of arousals is not only diagnostically relevant but prognostically critical in high-risk populations like AF.

Recent data from a large prospective cohort reinforce the pathophysiological relevance of sleep fragmentation per se in the development of AF. Wang et al. 2025 demonstrated that markers of disturbed sleep continuity, such as increased arousal index, prolonged wake after sleep onset, and reduced sleep efficiency, were independently associated with higher odds of incident AF, in individuals without OSA. A potential mechanism could be that sleep disruption promotes systemic inflammation (e.g., increased high-sensitivity C-reactive protein, TNF, and IL-6), leading to atrial fibrosis and increased heterogeneity in atrial conduction. In addition, oxidative stress and autonomic imbalance may further exacerbate arrhythmogenic remodeling. These findings suggest that even in the absence of clinically defined OSA, disrupted sleep architecture exerts adverse electrophysiological effects, highlighting the importance of recognizing cortical arousals as not only diagnostic markers but potentially modifiable contributors to AF risk [18]. On the other hand, besides OSAS, growing evidence shows a strong link between insomnia and both the high prevalence and increased risk of cardiovascular diseases [19]. Insomnia, likely through mechanisms of physiological and cognitive hyperarousal, may act as a trigger for atrial fibrillation episodes [20] and has been identified as an independent risk factor for post-ablation recurrence of AF [21, 22]. Given its high prevalence in this population and its potential pathophysiological role, the comprehensive evaluation of sleep disorders is crucial to accurately characterize sleep architecture, including arousals and respiratory events.

Despite the recognized impact of timely diagnosis and treatment of OSAS on AF-related outcomes, underdiagnosis remains a significant clinical concern. Although the AASM advises against the use of HSAT in patients with cardiovascular disease, including those with AF, these simplified tests are still frequently employed in clinical practice, due to their accessibility, cost, and feasibility in routine clinical settings. However, HSAT lacks the capacity to detect arousals and assess sleep architecture, limiting its diagnostic accuracy in this population. Our findings reinforce that type III devices, although widely used, may underperform in this population. Previous studies have shown that reliance on respiratory parameters alone can lead to misclassification of OSAS severity and underdiagnosis in symptomatic but non-desaturating patients [23, 24].

Won et al. (2020) also highlighted the significance of assessing how factors such as desaturations, arousals, sleep stages, and body position affect the AHI [22]. Furthermore, they stressed that defining the physiological endotypes of OSA is crucial for achieving a more comprehensive understanding of the disorder and for predicting clinical outcomes.Hence, PSG remains the gold standard for accurate diagnosis and severity characterization in this subgroup. More accurate classification of OSAS severity through arousal-inclusive scoring has therapeutic implications. It can influence therapeutic pathways, including CPAP titration thresholds and inform rhythm control strategies [25]. Evidence suggests that effective OSAS management improves AF ablation success rates and reduces arrhythmia recurrence. Thus, underestimating disease severity due to incomplete scoring criteria may compromise the overall effectiveness of AF management [26, 27]. The incorporation of standardized protocols for identifying sleep-disordered breathing in AF patients is strongly recommended. Sousa et al. described a multidisciplinary clinical pathway designed to facilitate the diagnosis of sleep-disordered breathing in patients with atrial fibrillation [28]. The recognized limitations of screening based solely on symptoms underscore the importance of utilizing objective assessments such as polysomnography, even among asymptomatic individuals.

### Limitations

This study has several limitations. First, the sample size was relatively small and drawn from a single tertiary cardiology center, which may introduce referral bias and limit generalizability. Second, the absence of a control group without OSAS precludes comparison of diagnostic performance across populations. Third, the lack of longitudinal follow-up prevents the evaluation of clinical outcomes such as rhythm control success, cardiovascular events, or hospitalizations. Moreover, arousal scoring was limited to EEG-detected cortical arousals; autonomic arousals were not assessed. Future studies with larger cohorts and prospective follow-up are warranted to validate these findings.

## Conclusion

OSAS is highly prevalent among AF patients, with most cases going undiagnosed. As AF is associated with increased stroke risk, reduced quality of life, and elevated mortality [29, 30], early identification of modifiable risk factors is essential for AF management, along with anticoagulation, rhythm and rate control. Current guidelines recommend routine OSAS screening in AF patients [29], yet commonly used diagnostic tools may fail to capture events relevant to arrhythmia pathogenesis, particularly in non-desaturating individuals. Our study highlights the added diagnostic and prognostic value of arousal-inclusive hypopnea scoring. A simplified, large-scale screening approach must balance accessibility with diagnostic precision, particularly in high-risk subgroups.

Incorporating arousal-based criteria should be viewed not as a mere technical adjustment, but as a clinically essential tool for accurate disease characterization and individualized management. In patients with AF, reliance on desaturation-only diagnostic models may lead to substantial underdiagnosis, with direct implications for treatment decisions and long-term outcomes.

## Data Availability

Data that support the findings of this study are not publicly available due to privacy reason but are available from the corresponding author upon reasonable request.

## References

[1] Stevenson IH, Teichtahl H, Cunnington D, Ciavarella S, Gordon I, Kalman JM (2008) Prevalence of sleep disordered breathing in paroxysmal and persistent atrial fibrillation patients with normal left ventricular function. Eur Heart J 10.1093/eurheartj/ehn214 18515807

[2] American Academy of Sleep Medicine (2024) The AASM Manual for the Scoring of Sleep and Associated Events: Rules, Terminology and Technical Specifications, Version 3.0

[3] Linz D, Kadhim K, Kalman JM, McEvoy RD, Sanders P (2019) Sleep and cardiovascular risk: how much is too much of a good thing? European Heart Journal, vol 40. Oxford University Press, pp 1630–1632 10.1093/eurheartj/ehy772 30517630

[4] Nalivaiko E, Catcheside PG, Adams A, Jordan AS, Eckert DJ, Mcevoy RD (2007) Cardiac changes during arousals from non-REM sleep in healthy volunteers. Am J Physiol Regul Integr Comp Physiol [Internet] 292:1320–1327 Available from: www.ajpregu.org 10.1152/ajpregu.00642.2006 17110530

[5] Sleep-related breathing (1999) Disorders in adults: recommendations for syndrome definition and measurement techniques in clinical research. The report of an American academy of sleep medicine task force. Sleep 22(5):667–689 10450601

[6] Berry RB, Budhiraja R, Gottlieb DJ, Gozal D, Iber C, Kapur VK et al (2012) Rules for scoring respiratory events in sleep: update of the 2007 AASM manual for the scoring of sleep and associated events. J Clin Sleep Med 08(05):597–619 10.5664/jcsm.2172 PMC3459210 23066376

[7] Shapira-Daniels A, Mohanty S, Contreras-Valdes FM, Tieu H, Thomas RJ, Natale A et al (2020) Prevalence of undiagnosed sleep apnea in patients with atrial fibrillation and its impact on therapy. JACC Clin Electrophysiol 6(12):1499–1506 33213809 10.1016/j.jacep.2020.05.030

[8] Desteghe L, Hendriks JML, McEvoy RD, Chai-Coetzer CL, Dendale P, Sanders P et al (2018) The why, when and how to test for obstructive sleep apnea in patients with atrial fibrillation. Clin Res Cardiol 10.1007/s00392-018-1248-9 29651786

[9] Mehra R, Benjamin EJ, Shahar E, Gottlieb DJ, Nawabit R, Kirchner HL et al (2006) Association of nocturnal arrhythmias with sleep-disordered breathing: the sleep heart health study. Am J Respir Crit Care Med 10.1164/rccm.200509-1442OC PMC2662909 16424443

[10] Asirvatham SJ, Kapa S (2009) Sleep apnea and atrial fibrillation. The autonomic link. J Am Coll Cardiol 54:2084–2086 19926017 10.1016/j.jacc.2009.09.017

[11] Todd K, McIntyre WF, Baranchuk A (2010) Obstructive sleep apnea and atrial fibrillation. Nat Sci Sleep 2:39–45 23616697 10.2147/nss.s7625 PMC3630930

[12] Tanaka N, Okada M, Tanaka K, Onishi T, Hirao Y, Harada S et al Sleep apnea severity in patients undergoing atrial fibrillation ablation: Home sleep apnea-test and polysomnography comparison. J Arrhythm [Internet]. 2023 Aug 1 [cited 2025 Apr 21];39(4):523–30. Available from: https://pubmed.ncbi.nlm.nih.gov/37560275/10.1002/joa3.12869 PMC10407183 37560275

[13] Riha RL, Celmina M, Cooper B, Hamutcu-Ersu R, Kaditis A, Morley A et al (2023) ERS technical standards for using type III devices (limited channel studies) in the diagnosis of sleep disordered breathing in adults and children. Eur Respir J 61(1):2200422 36609518 10.1183/13993003.00422-2022

[14] Kapur VK, Auckley DH, Chowdhuri S, Kuhlmann DC, Mehra R, Ramar K et al (2017) Clinical practice guideline for diagnostic testing for adult obstructive sleep apnea: an American academy of sleep medicine clinical practice guideline. J Clin Sleep Med 10.5664/jcsm.6506 PMC5337595 28162150

[15] Linz D, Linz B, Hohl M, Böhm M Atrial arrhythmogenesis in obstructive sleep apnea: Therapeutic implications. Sleep Med Rev [Internet]. 2016 Apr 1 [cited 2025 Jun 18];26:87–94. Available from: https://pubmed.ncbi.nlm.nih.gov/26186892/10.1016/j.smrv.2015.03.003 26186892

[16] Hindricks G, Potpara T, Dagres N, Arbelo E, Bax JJ, Blomström-Lundqvist C et al (2021) 2020 ESC guidelines for the diagnosis and management of atrial fibrillation developed in collaboration with the European association for Cardio-Thoracic surgery (EACTS). Eur Heart J 42(5):373–498 32860505 10.1093/eurheartj/ehaa612

[17] Shahrbabaki SS, Linz D, Hartmann S, Redline S, Baumert M (2021) Sleep arousal burden is associated with long-term all-cause and cardiovascular mortality in 8001 community-dwelling older men and women. Eur Heart J 42(21):2088–2099 33876221 10.1093/eurheartj/ehab151 PMC8197565

[18] Wang L, Hui X, Huang R, Xiao Y (2025) The association between sleep fragmentation and incident atrial fibrillation: a prospective cohort study in a community population. J Clin Sleep Med [Internet]. Mar 24 [cited 2025 Jun 20]; Available from: https://pubmed.ncbi.nlm.nih.gov/40123542/10.5664/jcsm.11614 PMC12225268 40123542

[19] Javaheri S, Redline S (2017) Insomnia and risk of cardiovascular disease, vol 152. Chest. Elsevier Inc, pp 435–444 10.1016/j.chest.2017.01.026 PMC5577359 28153671

[20] Gaffey AE, Rosman L, Lampert R, Yaggi HK, Haskell SG, Brandt CA et al (2023) Insomnia and early incident atrial fibrillation: A 16-Year cohort study of younger men and women veterans. J Am Heart Assoc.;12(20) 10.1161/JAHA.123.030331 PMC10757545 37791503

[21] Li Rbin, Zhang J dong, Cui X ran, Cui W (2024) Insomnia is related to long-term atrial fibrillation recurrence following radiofrequency ablation. Ann Med.;56(1) 10.1080/07853890.2024.2323089 PMC10906119 38423515

[22] Won CHJ, Reid M, Sofer T, Azarbarzin A, Purcell S, White D et al (2020) Sex differences in obstructive sleep apnea phenotypes, the multi-ethnic study of atherosclerosis. Sleep.;43(5) 10.1093/sleep/zsz274 PMC7215264 31687772

[23] Mulgrew AT, Fox N, Ayas NT, Ryan CF Diagnosis and initial management of obstructive sleep apnea without polysomnography: A randomized validation study. Ann Intern Med [Internet]. 2007 Feb 6 [cited 2025 Jun 18];146(3):157–66. Available from: https://pubmed.ncbi.nlm.nih.gov/17283346/10.7326/0003-4819-146-3-200702060-00004 17283346

[24] Kuna ST, Gurubhagavatula I, Maislin G, Hin S, Hartwig KC, McCloskey S et al (2011) Noninferiority of functional outcome in ambulatory management of obstructive sleep apnea. Am J Respir Crit Care Med [Internet]. May 1 [cited 2025 Jun 18];183(9):1238–44. Available from: https://www.atsjournals.org/doi/pdf/https://doi.org/10.1164/rccm.201011-1770OC?download=true 10.1164/rccm.201011-1770OC 21471093

[25] Zinchuk A, Edwards BA, Jeon S, Koo BB, Concato J, Sands S et al (2018) Prevalence, associated clinical features, and impact on continuous positive airway pressure use of a low respiratory arousal threshold among male united States veterans with obstructive sleep apnea. J Clin Sleep Med 14(5):809–817 29734986 10.5664/jcsm.7112 PMC5940432

[26] Maan A, Mansour M, Anter E, Patel VV, Cheng A, Refaat MM et al (2015) Obstructive sleep apnea and atrial fibrillation. Crit Pathways Cardiology: J Evidence-Based Med 14(2):81–85 10.1097/HPC.0000000000000044 26102018

[27] Naruse Y, Tada H, Satoh M, Yanagihara M, Tsuneoka H, Hirata Y et al (2013) Concomitant obstructive sleep apnea increases the recurrence of atrial fibrillation following radiofrequency catheter ablation of atrial fibrillation: clinical impact of continuous positive airway pressure therapy. Heart Rhythm 10(3):331–337 23178687 10.1016/j.hrthm.2012.11.015

[28] Sousa S, Cunha PS, Oliveira M, Bugalho A, Linz D, Drummond M (2023) Structured Pathway for Sleep-Disordered Breathing Screening in an Arrhythmia Unit Corresponding Author [Internet]. Vol. 16. Available from: www.jafib-ep.comwww.jafib-ep.com

[29] Joglar JA, Chung MK, Armbruster AL, Benjamin EJ, Chyou JY, Cronin EM et al (2024) 2023 ACC/AHA/ACCP/HRS Guideline for the Diagnosis and Management of Atrial Fibrillation: A Report of the American College of Cardiology/American Heart Association Joint Committee on Clinical Practice Guidelines. Vol. 149, Circulation. Lippincott Williams and Wilkins; pp. E1–156 10.1161/CIR.0000000000001193 PMC11095842 38033089

[30] Kamel H, Okin PM, Elkind MSV, Iadecola C (2016) Atrial Fibrillation and Mechanisms of Stroke: Time for a New Model. Vol. 47, Stroke. Lippincott Williams and Wilkins; pp. 895–900 10.1161/STROKEAHA.115.012004 PMC4766055 26786114

